# An Efficient Wireless Sensor Network for Industrial Monitoring and Control

**DOI:** 10.3390/s18010182

**Published:** 2018-01-10

**Authors:** Juan Aponte-Luis, Juan Antonio Gómez-Galán, Fernando Gómez-Bravo, Manuel Sánchez-Raya, Javier Alcina-Espigado, Pedro Miguel Teixido-Rovira

**Affiliations:** 1 OnTech Security LLC, C/Hispano Aviación, 7-9, 41300 Sevilla, La Rinconada, Spain; juan.aponte@ontech.es (J.A.-L.); javier.alcina@ontech.es (J.A.-E.); pedro.teixido@ontech.es (P.M.T.-R.); 2 Department Electronic Engineering, Computers, and Automatic, University of Huelva, Ctra Huelva-La Rábida, s/n, 21819 Huelva, Spain; fernando.gomez@diesia.uhu.es (F.G.-B.); msraya@diesia.uhu.es (M.S.-R.)

**Keywords:** remote industry monitoring, wireless sensor network, low power consumption

## Abstract

This paper presents the design of a wireless sensor network particularly designed for remote monitoring and control of industrial parameters. The article describes the network components, protocol and sensor deployment, aimed to accomplish industrial constraint and to assure reliability and low power consumption. A particular case of study is presented. The system consists of a base station, gas sensing nodes, a tree-based routing scheme for the wireless sensor nodes and a real-time monitoring application that operates from a remote computer and a mobile phone. The system assures that the industrial safety quality and the measurement and monitoring system achieves an efficient industrial monitoring operations. The robustness of the developed system and the security in the communications have been guaranteed both in hardware and software level. The system is flexible and can be adapted to different environments. The testing of the system confirms the feasibility of the proposed implementation and validates the functional requirements of the developed devices, the networking solution and the power consumption management.

## 1. Introduction

Wireless sensor networks (WSN) have emerged as one of the most promising technologies for the future. Some of the potential application domains are healthcare, army, environment and agriculture, industry, transportation systems and security [1,2,3,4,5,6,7,8,9,10]. Unlike other networks, WSN are designed for specific applications, and thus, they must satisfy a set of requirements that differs from one application to another. Therefore, researchers must address a variety of challenges to facilitate the widespread deployment of WSN technology in real-world domains. As a result, great efforts have been devoted to overcome the energy-saving problem and reliability [11,12].

Industrial plants integrate sensors connected to the control station through wire and wireless methods for continuous sensing and monitoring the status of the system. In this context, wireless technology gives a suitable support to the industry offering advantages in terms of low installation cost, scalability, flexibility, lack of cabling, intelligent-processing capability, high mobility and ease of deployment compared to conventional wired solutions. These advantages are promising for industry, where a considerable growth in the near future is expected [13].

Nevertheless, the use of WSN sensors for industrial applications requires accomplishing a set of hard constraints. For instance, monitoring and control of a specific process, demands the development of particular network architectures, mechanisms and algorithms that guarantee a high communication quality and reliability of the system [14,15]. In addition, as environmental conditions may be hard, industry implementations must ensure data reliability at all times. Finally, the design of an industrial communication network must ensure the availability of the data, authenticity and confidentiality [16].

It is not easy for the WSN designer to select the efficient solutions that should be considered in the design of application-specific WSN architectures. Although is difficult to propose a general approach for developing WSNs for industrial applications, this work considers some of the more common resource constraints of a WSN in this field related to: limited power consumption, robustness, processing capacity and storage.

The main contribution of the paper is related to the development of an efficient architecture in terms of power consumption and reliability, which involves both hardware and software design strategies. Moreover, the deployed WSN is easily scalable up. Authors have experimentally demonstrated the proposal feasibility and the energy efficiency of their approach.

The research covers several areas going from physical layer optimization to network layer solutions. IEEE 802.15.4 standard was chosen for communications because in the last few years it has become a reference for wireless applications with low data rates and high energy efficiency [17,18,19,20,21,22,23]. In fact, most of the WSN technologies are established by adapting IEEE 802.15.4 standard by using different frequencies and protocols. This standard, specifically designed for LR-WPAN networks, is a low-complexity protocol that offers low cost and energy features, as well as high versatility for the development of different topologies. We have devoted efforts to develop a specific stack since industrial applications require fast and timely responsiveness. The network architecture is particularly designed to address these industrial requirements.

The proposed system can be easily scaled up equipping various sensors on the designed sensor node or even including new sensor nodes to measure other physical magnitudes or parameters of interest. The wireless transmission was used to integrate all independent sensor signals that allowed for centralization and real-time control. Central management saves labor, increases efficiency and greatly reduces costs in industrial safety.

A particular case of study has been considered in order to validate the authors’ theoretical hypothesis. An industrial WSN for detecting and monitoring different types of gases has been implemented. The article gives details about the design of the modules and highlights the implementation of the theoretical approaches.

The paper is organized as follows: in the next section the components of the proposed architecture are presented and the general way of working is described. Section 3 is devoted to explaining the main features of the net by giving details about the communication protocol and the role played by the different elements of the network. Finally, Section 4 illustrated the experimental validation of the elements implemented for the case of study. Finally, conclusions are given in Section 5.

## 2. System Description and Operation

A WSN consists of tiny sensor devices spatially distributed forming a low level self-organized network, which send the data gathered through wireless links to sink nodes. Then, the sink nodes process or analyze the data locally or are connected to other networks, realizing real-time monitoring and control. Usually, the intrinsic characteristics of the wireless sensor network present new challenges in hardware design, communication protocols and application design. This requires modifying legacy protocols for conventional wireless networks or designing new effective communication protocols and algorithms.

**Figure 1:**
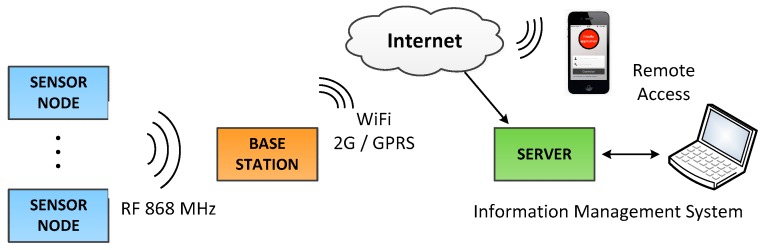
Architecture of the designed wireless sensor network.

Figure 1 shows the three different subsystems of the proposed industrial WSN architecture: The sensor devices or motes, the base station and the information management system.

Despite of being a common structure, each of the elements depicted in this figure requires a particular consideration for achieving the efficiency demanded by industrial applications. This section is devoted to describe the design of these elements in terms of: reliability, power consumption, data availability, authenticity and confidentiality, friendly and easy remote configuration. Theoretical approaches are first described and later turned into a real development by considering the proposed case of study: an industrial WSN for monitoring and detecting different types of gases.

### 2.1. Sensor Node

Several questions have been addressed in the design of the sensor nodes, concerning energy efficiency, reliability and latency reduction.

On the one hand, sensor nodes are designed to built a flexible solution and avoid complex cabling. Moreover, defining an energy efficient operation mode is essential for the target application. For this purpose, sensor nodes gather the data periodically but they alternate between working cycles (when the node is awake) and low power consumption cycles (when the node is slept). This issue allows the sensor nodes to keep the energy consumption to a minimum, extending their functionality for a reasonable period of time.

This operation mode together with an energy efficient protocol (described in detail in Section 3) has been proposed to save energy and guarantee that the data is reliably delivered to the sink node. To this aim, sensor nodes are deployed with a cluster-tree topology. Both the employed network topology and the protocol allow the designed system to increase its scalability, containing a large number of sensor nodes. In this way, low cost and small-sized sensor nodes have been implemented. Finally, latency has also been taken into account for practical operation. Latency is an important factor for the system reliability such as in the case of emergency response, and accuracy of data reporting in case of high frequency periodical data updates.

**Figure 2:**
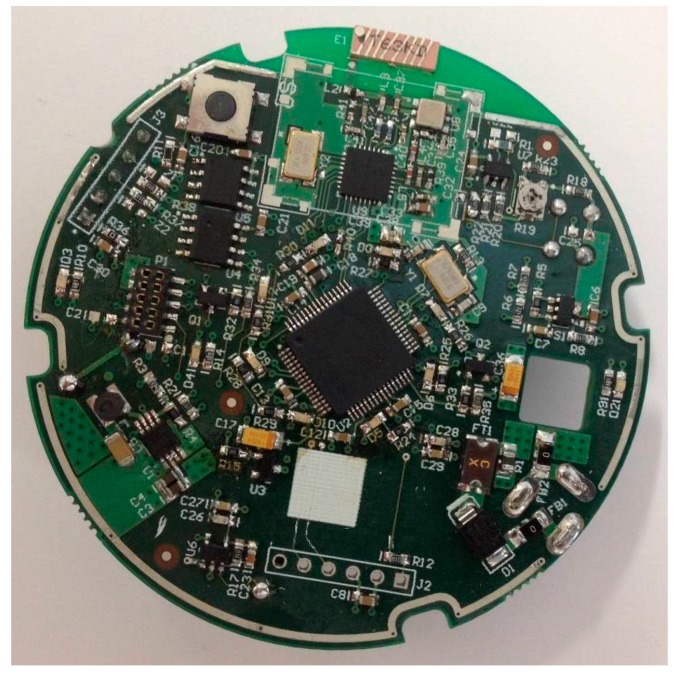
Gas sensing node.

All these ideas have been reflected in the design of the sensor implemented in the case of study. Figure 2 shows the board of the developed sensing node. The designed hardware consists of an analog gas sensor, a data processing module (microcontroller), a wireless communication module (wireless transceiver) and an energy module. The gas sensor comprises of all circuitry necessary for the detection of butane, methane and propane. The detection system is completed including a carbon monoxide (CO) sensor. Both the gas sensor and the CO sensor are placed on the bottom of the board.

The analog gas sensor TGS6810 from Figaro (Arlington Heights, IL, USA) has been chosen. This is a compact sensor whose stability, quick response and linear output makes it suitable for detecting combustible gases. The signal-conditioning circuit is basically a Wheatstone bridge, whose differential output voltage is converted to a single-ended signal and amplified by a non-inverting amplifier stage. The high sensitivity of this sensor can detect very small gas leaks in very early stages, avoiding accidents due to explosions, fires or inhalations. Regarding the CO sensor, the TGS5342 also from Figaro (Arlington Heights, IL, USA) has been used. This sensor generates a small current, linear to CO concentration, which is converted into an output voltage by an op-amp/resistor combination. The output signal of the analog sensors is converted to a binary value using the analog to digital converter of the microcontroller.

### 2.2. Base Station

The core of the proposed architecture is the base station which centralizes all system signals. This node is also known as gateway or sink node. It coordinates the network, receives the data from the sensor nodes, and processes and stores it into the information management system. For this purpose, the base station has to include several elements responsible for performing these main tasks. First of all, an efficient wireless circuit for implementing communication with the sensor nodes is needed. Secondly, a redundant communications system that supports outer communications has to be included. The base station should also provide a simple human interface to allow basic operation. Finally, although the base station is usually plugged to a power supply, it is also subject to energy constraint and the possibility of being powered by a battery has to be taken into account.

In this article a general purpose design for a base station is proposed. It can be applied either for the considered case of study or for another industrial application. In the following the proposed design is described in detail.

**Figure 3:**
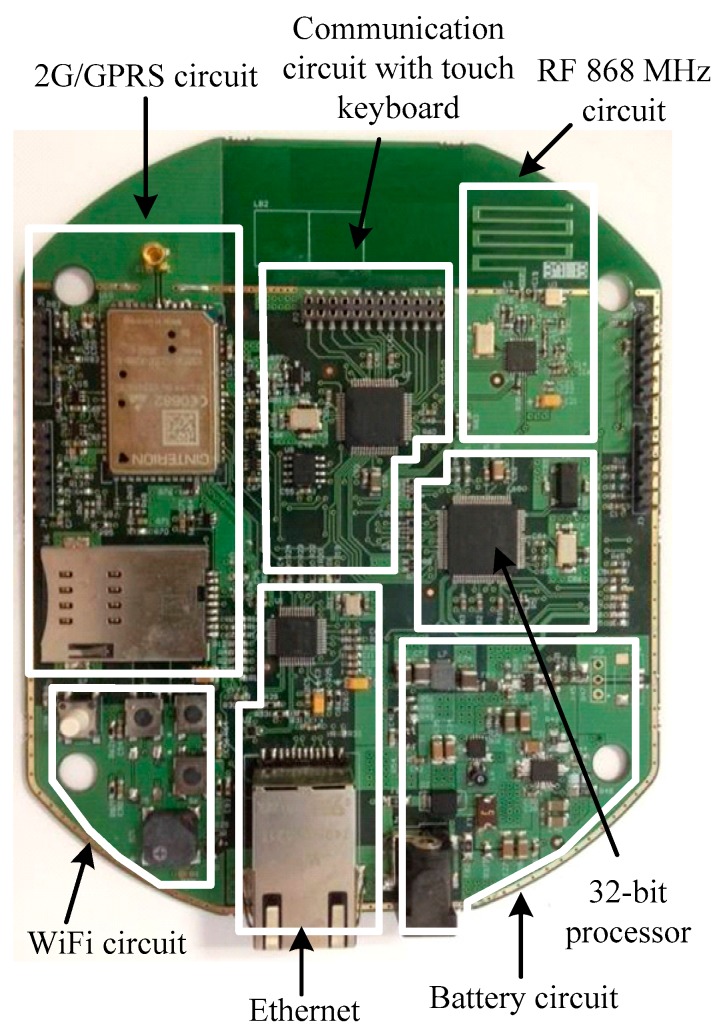
Image of the main board of the base station.

The hardware of the base station is divided into two printed circuit boards. The primary board is the core of the base station, and includes the following elements: a RF (radio frequency) communication circuit compatible with the one used by the sensor nodes, a power supply system (internal battery, battery charger), an outer communication system, control circuits and data processing (MCU). Figure 3 shows details of the hardware implementation of this main board.

**Figure 4:**
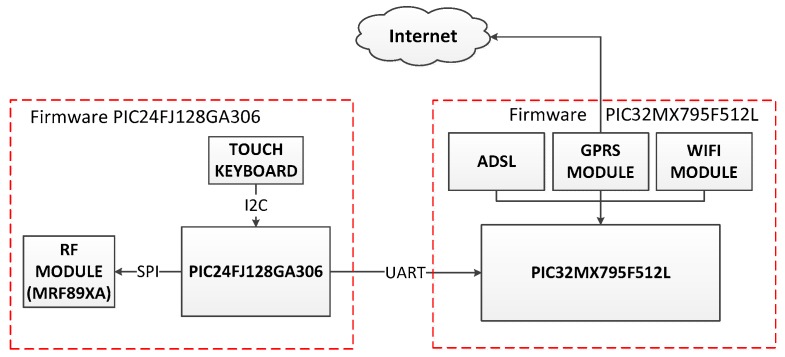
Block diagram of the firmware structure of the base station.

The base station is structured in a multi-processor design with two independent control circuits as shown in Figure 4: One based on the 16-bit PIC24FJ128GA306 microcontroller and another based on the 32-bit PIC32MX795F512L microcontroller, both from Microchip (Chandler, AZ, USA). These microcontrollers meet all needed specifications, and the main tasks assigned to each are as follows: the 16-bit microcontroller handles the control of the capacitive touch keyboard and its Light Emitting Diode (LEDs), Serial Peripheral Interface (SPI) communications with the RF 868 MHz circuit for the wireless data transmission with the sensor nodes, and communications with the 32-bit microcontroller in order to synchronize with the external world all events occurring in wireless peripherals attached to the system. The 32-bit microcontroller performs two basic tasks: managing the main firmware, and controlling the outer communication system. These communications can be supported by three different technologies (Ethernet, Wi-Fi, and 2G/GPRS modules). By means of these mechanisms the base station is able to retransmit the information gathered by the sensor nodes to the external world. Thus, the versatility of the base station ensures a feasible communication with the server and Internet through wired and wireless connections. The communication is further assured with the 2G/GPRS module for losses of Ethernet and Wi-Fi connections, making the system more robust and even avoiding sabotages.

The short-range radio module exchanges data via wireless at 868 MHz following a specific stack, which has been developed conciliating the most appropriate features of IEEE 802.15.4 and MiWi for the target application. Additionally, encryption algorithms have been implemented to improve the security of the RF communications, as will be discussed later. An electromagnetic shield to avoid interference problems between the RF circuit and adjacent circuits has also been included.

**Figure 5:**
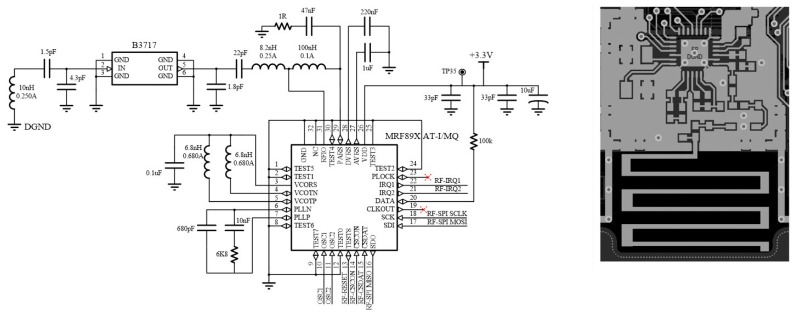
Designed antenna and scheme of the radio frequency (RF) module.

The radio module is based on the transceiver MRF89XA from Microchip (Chandler, AZ, USA), chosen due to the very low power consumption and for being particularly suitable for the sensor nodes. A low cost solution has been chosen designing a PCB trace antenna, avoiding the use of conventional antennas or commercial elements that would add cost to the device. Figure 5 shows the designed antenna and the scheme of the RF module.

**Figure 6:**
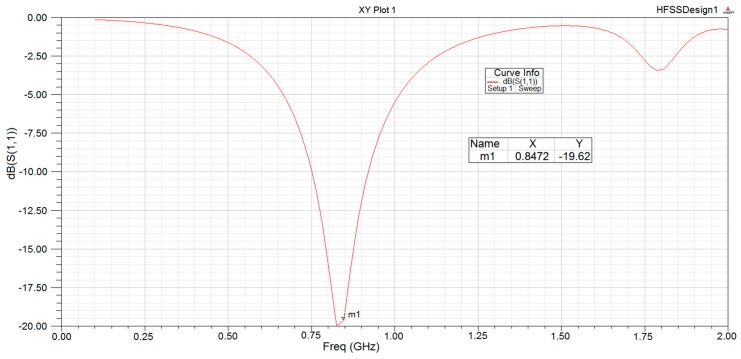
Simulated performance of the antenna.

Simulation software for high performance antennas has been used. Results shown in Figure 6 guarantee the proper operation.

The base station is plugged to a power supply but it can also be supplied by a battery. Additional circuitry has been included based on the integrated linear charger bq24070 from Texas Instruments (Dallas, TX, USA) as a system power-path management device. This combined approach allows the base station to be simultaneously powered while the battery is charging, and avoids charge-discharge cycles, greatly extending the battery life. The base station is able to continue working properly for an additional period of 10 hours, if an electrical power outage occurs.

**Figure 7:**
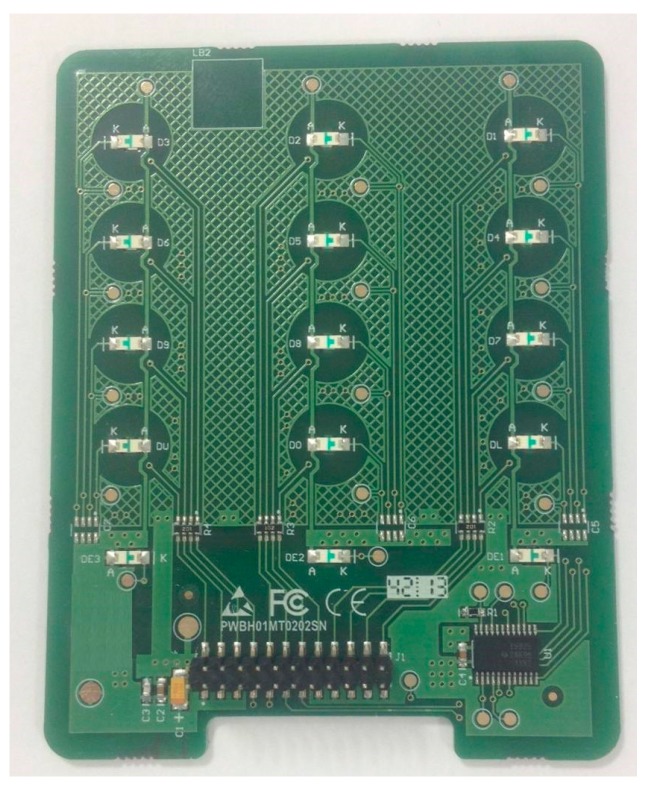
Touch keyboard of the base station.

The secondary board of the base station includes a capacitive touch keypad with associated electronics and LEDs as shown in Figure 7. The design of this board and the integration in the base station has been a challenge, as the capacitive sensing technology to detect one touch (only functional with a human finger), with the proper choice of the materials which permit the transmission of signals from the user’s finger to the sensor of the board (copper pad), had to be merged. For the design of this board, the capacitive keyboard inputs that are available in the 16-bit microcontroller were initially used. After stringent preliminary tests, random pulses occurred due to external noise and the sensitivity was not entirely correct. Thus, the drivers CAP1166 with capacitive sensors especially suitable for these applications were used, providing satisfactory results after repeating the tests. The inclusion of the touch keyboard on the base station increases the versatility of the system. Although the system is controlled remotely, the capacitive touch keypad also allows the user to manually enter codes to enable or disable different parts of the system.

### 2.3. Information Management System

Several system status data need to be defined and gathered together with the sensor data. Moreover, it is important to remotely adjust system configurations and update and upgrade software programs. In addition, remote monitoring of system status is useful for system development, debugging, and maintenance purposes.

The information management system includes a web application and mobile applications for iOS and Android, which allow the control of all system parameters, and monitoring alarms and events. The web application allows configuring the system from a remote computer via Internet by authorized users, accessible through the outer communication system. The mobile application also provides alerts in real-time.

The complete monitoring and control for industry applications involves the measurement of several physical magnitudes to improve the quality of the processes. The designed wireless system is scalable, and thus the number of quantities to be measured is easily expandable.

## 3. Network Management

### 3.1. Preliminary Considerations

Initially we started from a known open source protocol proposed by the transceiver’s manufacturer, called MiWi. This is a low cost solution for applications where low power consumption, few computational resources and low data rates are required. It is based on the IEEE 802.15.4 standard, specifically designed for LR-WPAN networks, which offers high versatility for the development of different topologies.

The preliminary tests performed to MiWi concluded that it was not sufficiently robust and stable for the target application. Specifically, it presents large latencies, which were also variable and never deterministic, it even sometimes blocked part of the hardware that needed immediate attention of the processor because MiWi makes intensive use of busy-wait cycles. Duty cycle is one of the most commonly used approaches to reduce the energy consumption during waiting time. Furthermore, it is one of the major sources of energy waste. Therefore, for the firmware of RF communications, we preferred to develop a specific stack. The main features of the developed stack are the following:

- SPI controller with an interrupt mechanism to minimize latency and the busy-wait cycle of the microcontroller
- Driver for the Microchip MRF89XA transceiver. The controller is ready to work in background, so it does not use busy-wait cycles, and therefore there is no latency
- A medium access (MAC) layer, which is responsible for the communication between nodes by routing through a unique fixed address, read from the Electrically Erasable Programmable Read Only Memory (EEPROM). This layer is also designed to minimize the busy-wait cycles and for most of the process is done in background, reducing latency as much as possible. For the design of this layer aspects of the IEEE 802.15.4, such as timeouts or frame formats were considered
- A logical link layer (LLC). This layer handles the logical addressing of the nodes, the allocation of roles within the network (PAN coordinator, coordinator or end device), assignment of the logical address (which is not fixed), route management, network discovery, traffic control and management of consumption modes. The design of this layer is also designed to minimize busy-wait times and latency
- AES128 encryption layer is the responsible for encrypting the messages that will be sent to the other devices. This layer improves the system safety
- Presentation layer. This layer (above the LLC layer) performs the functions of an interface between the stack and the node. It is responsible of configuring the stack, and of coordinating its operation
- Driver for EEPROM. It is responsible of EEPROM access without interfering with the radio module. This prevents a high coupling between them, since both may operate asynchronously, sharing the same SPI bus.

### 3.2. Protocol Description

The designed stack was performed in layers so that they can be used independently and disengaging the different levels of functionality, allowing an enhanced code, portability, adaptation to new hardware and better debugging and testing. The same addressing scheme proposed by MiWi and supported for cluster tree network topologies (provided by the LLC layer) have been used in the new approach.

The main aim of the design is to minimize the latency and the processor usage almost without busy-wait cycles. Thus, the entire design is based on the use of interruptions by the SPI controller. All functions are implemented as state machines in which each state is triggered by an interruption when the previous operation ends.

The communication of asynchronous events, such as the reception of new messages or network outage is performed through a set of callbacks added to the APIs of each layer. This structure (although the development has been somewhat more complex than in the case of stacks as in MiWi) has allowed to reduce the maximum processor usage, and the stack scarcely influences the other components of the nodes. The power consumption is also reduced because the CPU is free a longer period of time.

MiWi, similar to the specifications defined by IEEE 802.15.4, supports three network frameworks: Star, Cluster Tree and Mesh; and defines two types of devices: full-function devices (FFD) and reduced-function devices (RFD) as shown in Table 1. The roles of the nodes can be divided into PAN Coordinator, Coordinator and End Device as shown in Table 2. Sensor nodes could act as a full function device (FFD) or reduce function device (RFD). In FFD, the nodes never sleep, as they need to relay the information from the RFD to the sink node, while a RFD will only wake after a certain period of time to: gather the sensor data, forward it to a FFD node and go to the sleep mode.

**Figure 8:**
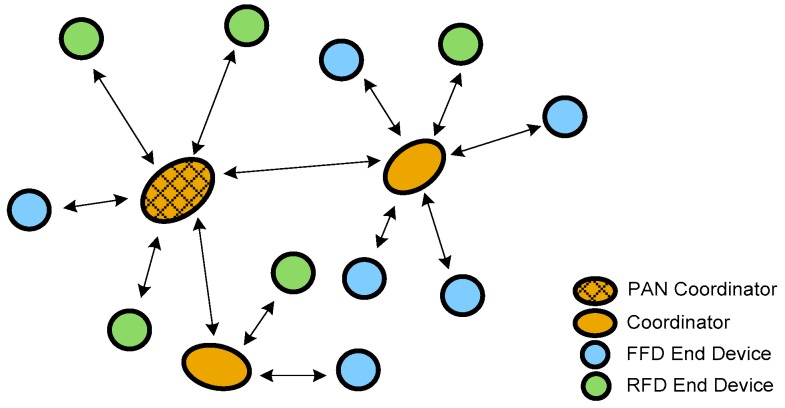
Cluster-tree network topology.

The sensor nodes must use low consumption operating modes in order to achieve good energy efficiency. For this purpose, a cluster-tree network has been deployed so that the sensor nodes (acting as End Devices) can be set as reduced-function devices (RFD) with the ability to enter in the sleeping mode in which the consumption can be considered negligible. Figure 8 shows the topology of a cluster-tree network. A PAN Coordinator node is responsible for managing the network. This PAN Coordinator assigns the addresses to the END Devices and to the other Coordinators. A coordinator node also assigns addresses to the END Device nodes located inside its transmission range. If a new node is switched on, it starts on the awake mode and takes contact with the FFD node inside its transmission range (either a Coordinator or the Pan Coordinator). Once the FFD node assigns the address, the END Device starts its cycle switching from awake to sleep mode. This mechanism facilitates the system scalability.

The medium access protocol that has been chosen corresponds to the IEEE 802.15.4 specification. The designed layer is located above the MAC layer and must specify the following mechanisms: (1) Assigning addresses to new nodes to be included in the network; (2) Determining the necessary elements to create a network topology based on a Cluster Tree topology; (3) Message routing inside the network, if several jumps are required to reach the destination; (4) Finding out when a node loses the connection with its coordinator; (5) Security configuration; (6) Configuring parameters for energy efficiency, such as transmission power, sensitivity and sleep states of the transceiver; (7) Preventing the loss of messages even if the target node is in sleep mode.

Security is critical when designing a wireless communication system. Intrusions may result in harmful or even disastrous situations in industrial plants. The designed network includes security mechanisms and message encryption to maintain data integrity and prevent interception of the transmitted data between the nodes of the network.

Regarding the flow control, an un-slotted CSMA/CA mechanism without beacons is used when a node wants access to the medium for message transmission, by means defined in IEEE 802.15.4.

### 3.3. Energy Conservation and Data Reliably Deliberation

The energy optimization can be made at different levels. As sensor nodes require efficient energy, optimized hardware for low power operation requires selectable power states (awake or sleep). As in sleep mode the transceiver is disabled, it is necessary to establish some sort of protocol to avoid the loss of messages.

As discussed above, only END Devices can enter sleep mode. The mechanism that prevents the loss of messages when END Devices are in sleep mode consists in making Coordinators store messages addressed to their END Devices. Once a node is awake it must explicitly request data to the Coordinators using Data Request commands defined by the IEEE 802.15.4 standard. Thus, we will also have a flow control for the case that an END Device takes too long to process a message before it can receive the following. The mechanism of energy management, which in principle only makes sense for the case of an END Device, allows controlling the way that they ask data to the Coordinators.

Obviously, the sink node requires high processing power to coordinate the whole network activity, thus, it requires the highest energy consumption amongst the devices.

A detailed power budget analysis of the transceiver was necessary to determine the capacity of the system. The power consumption is determined by measuring the current draw and the time spent in each of its operating modes. The tests were carried out for four functional states: Data transmission, acquisition, listening and sleep mode, for a transmission power of 13 dBm. The measured power consumption was equal to 12.8 mA for the transmission state, 6.52 mA for acquisition, 2.99 mA for listening and 17 µA for sleep state. The mean elapsed time used in receiving and answering the pending messages, measured between the awakening of the node and the return to sleep mode, was 140 µs (wakeup time). The mean current consumption for the transceiver was limited to 150 µA over a certain period of time. Thus, the following expression must be met:

(1) $$F_{tw}\cdot I_{w}+F_{ts}\cdot I_{s}\leq I_{\max}$$

where *F_tw_* is the fraction of time that the transceiver is turned on, and *I_w_* is the current consumption of the transceiver in that state; whilst *F_ts_* is the fraction of time that the transceiver is in sleep mode, and *I_s_* is the current consumption in this same state. As *F_ts_ = (1 ­ F_tw_)*, the above expression can be rewritten, yielding,

(2) $$F_{tw}\leq \frac{I_{\max}-I_{s}}{I_{w}-I_{s}}$$

Substituting the measured current values, *F_tw_ ≤* (150 µA − 17 µA)/(12.8 mA − 17 µA) ≅ 0.0104. Considering the power consumption per hour, the time that the transceiver can remain in active mode is T_w_ = 0.0104 × 3600 s = 37.45 s. Thus, the transceiver allows accomplishing a very high number of requests per hour (37.45 s/140 µs = 2.67 × 10^5^). Therefore, at maximum power, the consumption of the transceiver allows a large number of requests to be made without breaking the time requirements.

Once the hardware was validated and correct operability was assured, the next step was to review the software incorporating low-consumption modes in all the motes.

**Figure 9:**
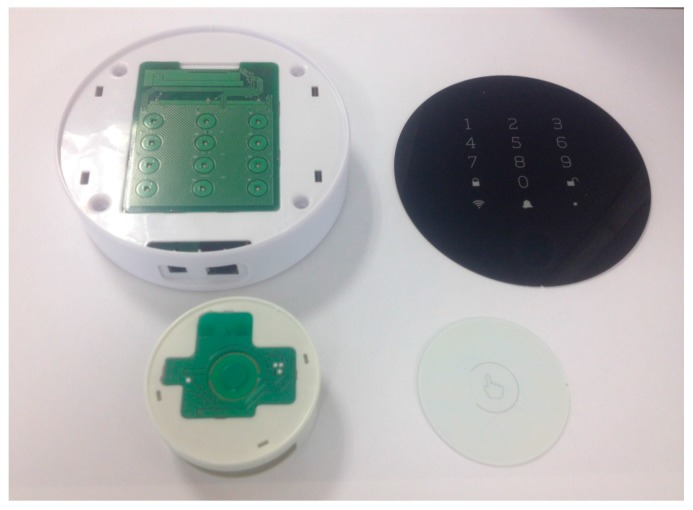
Image of the base station and gas sensor, and packing design.

Figure 9 shows the final implementation of the base station and the gas sensor, and the packing designed to protect them. The final dimensions of the sensor packing are 70 mm in diameter with a height of 30 mm. For the base station they are 140 mm in diameter and 35 mm in height.

## 4. Experimental Validation: Monitoring and Control Application

Considering the case of study, web and mobile applications that implement a graphical user interface have been developed. By means of this software, real time monitoring of physical magnitudes and battery level of all active nodes can be done. Furthermore, all information measured by the nodes can be preserved for further processing. This information allows the user to act on the system at any time.

**Figure 10:**
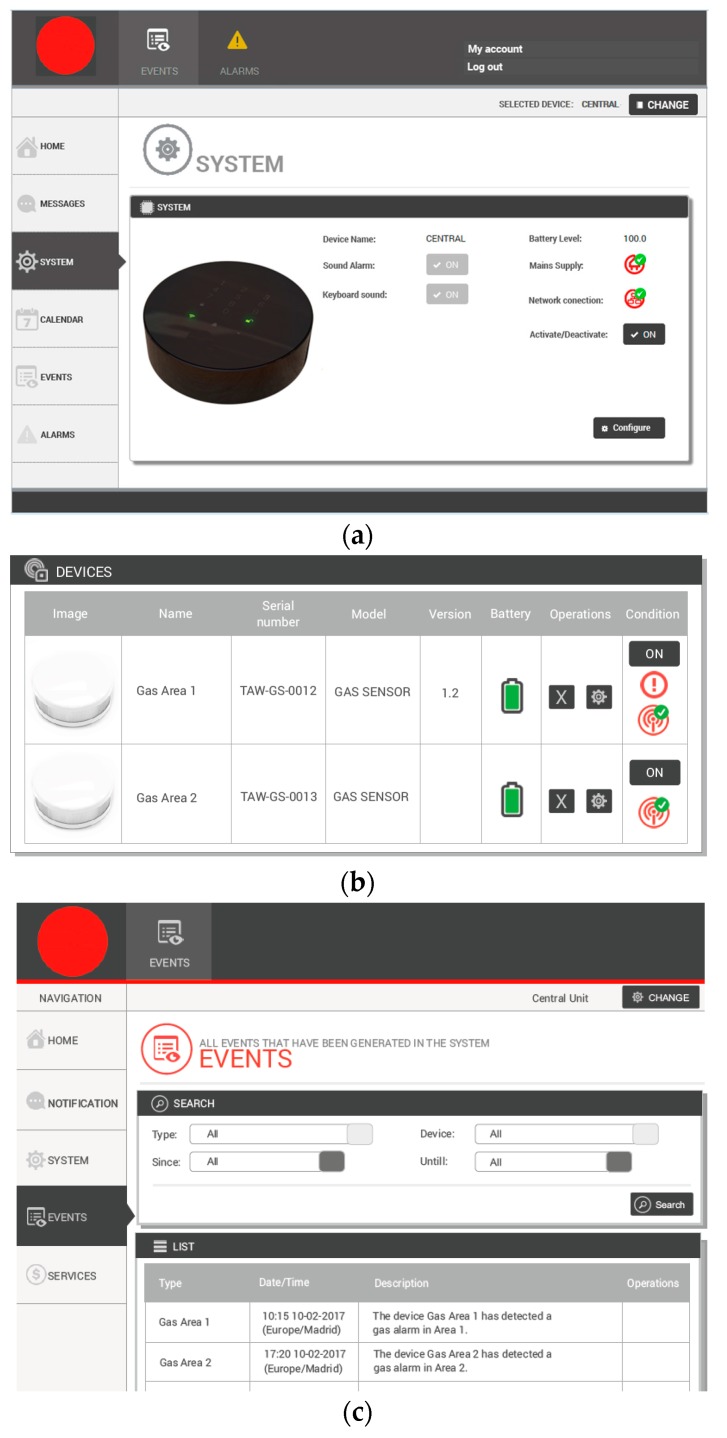
Main views of the monitoring application.

The web and the mobile applications allow the user to configure all devices of the wireless system, as well as monitoring and receiving alarm warnings. Figure 10 shows the main views of the remote monitoring and control application, and the main features are as follows:

1. Registration and access to the control panel. Users may register through the web portal, which includes their user data and credentials. Once inside the application, the different system devices can be configured and monitored
2. The battery voltage level is closely monitored as a part of the remote system status monitoring service. Near-real-time monitoring of such a system status data is important in determining battery efficiency and early detection of severe battery degradation to prevent system failure and the loss of data
3. Methods of notification about alarms and events provided by the system (Short Message Service (SMS), Email and mobile notifications) can be changed as well as selecting which user should receive such notifications
4. The configuration of the base station and the peripherals that comprise the system allows the user to activate them, disable them, or make changes in the configuration of each device, such as changing detection thresholds, detection periods, etc.
5. Event monitoring. The various events generated in the system can be filtered and listed. These events refer to changes in the configuration of devices and accesses to the system
6. Alarm monitoring. The various alarms generated in the system can be filtered and listed.

The sensors proved to be robust and have very high performance. The gas sensor meets the UNE-EN 50194 standard, and enables the detection of toxic and explosive gases, such as, butane, propane, methane, town gas, natural gas and others. The gas detector is calibrated to detect the presence of gases at a level of concentration of 10% L.E.L. (Lower Explosion Limit), corresponding to: 5.000 p.p.m of methane, 2.000 p.p.m. of propane and 1.800 p.p.m. of butane following the mentioned standard.

**Figure 11:**
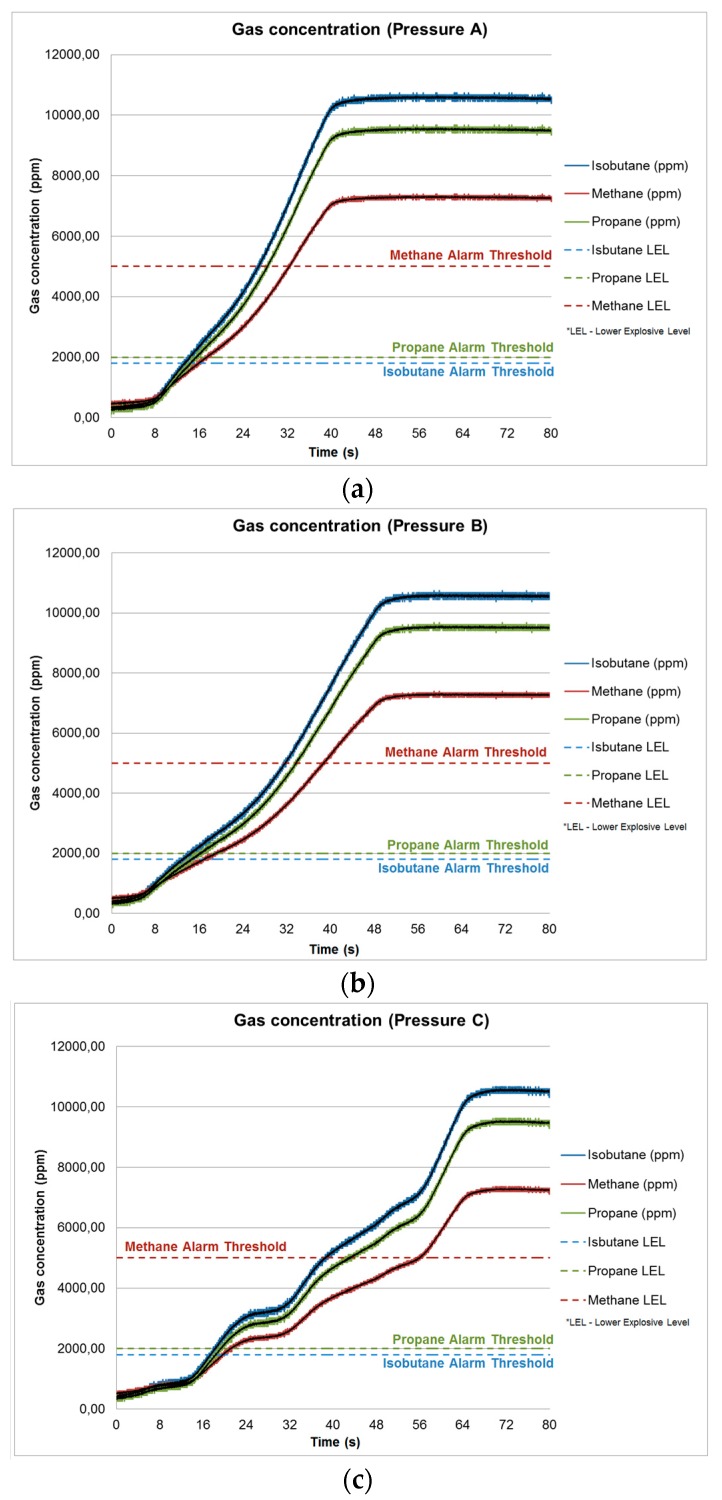
Time response of gas detector measurements in three cases. (**a**) Higher pressure, (**b**) Middle pressure and (**c**) Lower pressure.

Figure 11 shows three cases of measured values for butane, methane and propane. Tests were performed in a room of dimensions 3 m × 5 m and 2.5 m of height. The gases were introduced at three different pressures A, B and C (pressure A is the highest and pressure C is the lowest). Then, the volume of gas in the room was measured for 80 seconds. Note that for the case of higher pressure (A) the gas sensor responds faster and the measured gas concentration is also higher. In the opposite situation is the case of lower pressure (C). When any of the above threshold levels (dashed lines) is exceeded, the detector activates an alarm indicator light for 5 seconds to confirm the detection and prevent false alarms. When this time is up, if the level of detection is maintained, the alarm for the mobile application is activated. When the detector, in alarm state, detects a gas concentration level of less than 10% L.E.L for a certain time, it deactivates the alarm indicator light and the alarm output for the mobile application.

## 5. Conclusions

This paper presents an original approach regarding the design of an efficient WSN for industrial monitoring and control in terms of power consumption, data availability, authenticity, confidentiality, and friendly and easy remote configuration. The proposal is aimed to accomplish industrial requirements, and thus, involves hardware and software design strategies for achieving robust nodes and reliability and security in the communications. A real WSN designed to measure industrial parameters has been implemented, so that experimental validations can be performed.

The implemented wireless system includes the development of a low cost gas sensor node with optimized size, a base station and a graphical user interface for remote access to industrial conditions and real-time control and monitoring. The system has been designed to be easily scaled-up to measure other parameters of interest. All aspects of the monitoring system such as sensing activity, transmission from sensor nodes to base station, remote transmission from the base station to the control center or information management system, data storage, and visualization, have also been designed and implemented. The system requires low maintenance and includes energy-budget management. The flexibility of the system allows being used to realize remote monitoring in coal mine, buildings, etc.

## Figures and Tables

**Table 1:** Class of devices by means of IEEE 802.15.4.

Device Class	Offered Services	Power Path	Low Power Configuration
Full Function Device (FFD)	All	Mains	Always ON
Reduced Function Device (RFD)	Limited	Batteries	Always in SLEEP

**Table 2:** Devices role (IEEE 802.15.4).

Device Role	Class of Device (IEEE)	Function in the Network
PAN Coordinator	FFD	Only one by network. Create network, assign addresses, and hold the connections table
Coordinator	FFD	Optional. Make bigger the range of the network and make possible to add more devices to the network. Control and monitoring functions
End Device	FFD o RFD	Control and monitoring functions
